# Amphiphilic Block Copolymers Bearing Hydrophobic γ-Tocopherol Groups with Labile Acetal Bond

**DOI:** 10.3390/polym12010036

**Published:** 2019-12-25

**Authors:** Shotaro Yukioka, Takuya Kitadume, Suchismita Chatterjee, Gan Ning, Tooru Ooya, Shin-ichi Yusa

**Affiliations:** 1 Graduate School of Engineering, University of Hyogo, 2167 Shosha, Himeji, Hyogo 671-2280, Japan; shotaro0326shotaro@gmail.com; 2 Graduate School of Engineering, Kobe University, 1-1 Rokkoudai, Nada, Kobe, Hyogo 657-8501, Japan; kitadume@gmail.com (T.K.); Ning@gmail.com (G.N.); ooya@tiger.kobe-u.ac.jp (T.O.); 3 Institute of Material Structure Science, High Energy Accelerator Research Organization, 1-1 Oho, Tsukuba 305-0801, Japan; suchichatterjee@gmail.com

**Keywords:** *γ*-tocopherol, acetal bond, amphiphilic diblock copolymer, polymer micelles

## Abstract

High concentrations of γ-tocopherol (γTCP) tend to show antioxidant, anti-inflammatory, and anticancer effects. In this study, we prepared polymer micelles under acidic conditions with a controlled release of γTCP due to the decomposition of pendant acetal bonds. First, a precursor diblock copolymer composed of poly(ethylene glycol) (PEG) and acrylic acid (AA) was prepared. This was followed by the synthesis of an amphiphilic diblock copolymer (PEG_54_-P(AA/VE6/γTCP29)_140_), incorporated into hydrophobic γTCP pendant groups attached to the main chain through an acetal bond. The prepared PEG_54_-P(AA/VE6/γTCP29)_140_ was further dispersed in water to form polymer micelles composed of hydrophobic cores that were generated from a hydrophobic block containing γTCPs and hydrophilic shells on the surface. Under acidic conditions, γTCP was then released from the core of the polymer micelles due to the decomposition of the pendant acetal bonds. In addition, polymer micelles swelled under acidic conditions due to hydration of the core.

## 1. Introduction

Amphiphilic diblock copolymers in water form polymer micelles consisting of a hydrophobic core and hydrophilic shell [1,2,3]. This serves as an effective route to encapsulate hydrophobic anticancer drugs into the core, thereby improving the solubility of the reagents while limiting side effects [4]. As such, the passive targeting of drug-loaded polymer micelles of 10–100 nm average size can enhance the permeability and retention (EPR) effect [5,6]. In general, polymer micelles with a size of several hundred nanometers are unable to penetrate normal vascular walls, while polymer micelles around tumor tissue can penetrate through defected vascular walls (enhanced permeability). This is due to incomplete neovascularization around the tumor and gaps that form between vascular endothelial cells. In addition, polymer micelles cannot be completely removed from the lymphatic tissue around the tumor due to the immaturity of the lymphatic tissue. Therefore, the leaked polymer micelles from tissue vesicular walls tend to accumulate around tumor tissue (enhanced retention). Such a property generated by the accumulation of polymer micelles around tumor tissue is the EPR effect [7]. However, in the passive targeting mechanism, the amount of drugs for delivery to the affected area is low, making the evaluation of the side effects of anticancer drugs non-negligible [8,9].

Recently, cancer-cell apoptosis has been reported using food-derived natural products [10,11]. γ-tocopherol (γTCP) is a type of vitamin E, contained in vegetable oils such as canola, soybean, and corn oils. This substance possesses antioxidative and anti-inflammatory effects, and demonstrates the potential to improve cardiovascular disease and prostate cancer [12,13]. According to the literature, an increase in γTCP concentrations in plasma may decrease the risk of prostate cancer [14]. However, it is well established that the accumulation of excess amounts of vitamin E in the body by a normal diet is impossible due to the low absorption rate of vitamin E that is in the range of 21%–29% [15]. Nevertheless, by local administration of more highly concentrated vitamin E, cancer-cell apoptosis can be induced without the use of anticancer drugs.

According to previous studies, the pH of cancer and healthy cells was determined as 5 and 7.4, respectively [16]. Acetal bonds under acidic conditions can also be readily cleaved into suitably functional moieties [17]. pH-responsive nanoparticles containing acetal linkage within the chemical structure for the controlled release of drugs under acidic conditions have been developed in the last two decades [18,19,20,21]. Simo et al. [22] reported that acetal linkage-containing hydrophilic *N*-(2-(tetrahydoro-2H-pyna-2-yl)oxy)ethyl acrylamide (HEAmTHP) was polymerized via reversible addition-fragmentation chain transfer (RAFT) radical polymerization using a hydrophobic poly(2-hydroxyethyl acrylate) (PHEA) macro-chain transfer agent (CTA) to prepare an amphiphilic diblock copolymer (PHEA-PHEAmTHP). In a subsequent study [23], the encapsulation of a hydrophobic antibiotic substance in the hydrophobic domain of formed PHEA-PHEAmTHP micelles in water was performed. Another study by Gold et al. investigated the effects of attached Amphotericin B to the the cell membrane of micro-organisms, which showed antibiotic property by breaking membrane structures [24]. Under acidic conditions, Amphotericin B was released from the polymer-micelle duet that cleaved the acetal linkages. Feng et al. [25] prepared hyperbranched polyesters (S-hbPE) containing multiple acetal linkages via condensation of 2,2′-(propane-2,2-diylbis(oxy)) diethanol, phosphoryl chloride, and poly(ethylene glycol) monomethyl ether (m-PEG_45_-OH). Chlorin e6 a photosensitizer for photodynamic therapy (PDT) was encapsulated into S-hbPE to prepare nanoparticles. Chlorin e6 was then released under acidic conditions around tumor tissue attributed to the decomposition of the acetal linkages. As expected, the release of chlorin e6 under acidic conditions around tumor tissue showed the high efficiency of PDT with reduced side effects. Thus, nanocarriers containing acid labile acetal linkages for delivery systems via endosome/lysosome possess advantages such as solubilization of hydrophobic guest molecules, and controlled release in the acidic conditions around tumor tissue.

**Figure 1:**
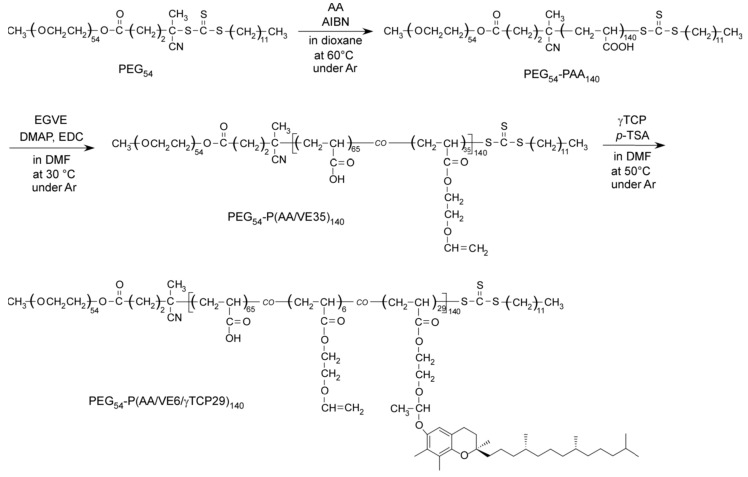
Synthesis of PEG_54_-PAA_140_, PEG_54_-P(AA/VE35)_140_, and PEG_54_-P(AA/VE6/γTCP29)_140_.

**Figure 2:**
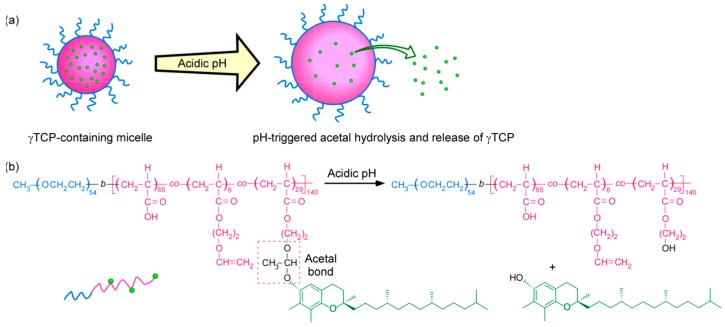
(**a**) Micelle formation and release of γTCP in acidic aqueous solution; (**b**) chemical structure of PEG_54_-P(AA/VE6/γTCP29)_140_.

In this study, we first prepared a precursor diblock copolymer (PEG_54_-PAA_140_) composed of biocompatible poly(ethylene glycol) (PEG) and poly(acrylic acid) (PAA) blocks. This was followed by the preparation of a diblock copolymer (PEG_54_-P(AA/VE35)_140_) via incorporation of vinyl ether (VE) groups to the pendant carboxylic acid groups of the PAA block through esterification. Finally, a diblock copolymer (PEG_54_-P(AA/VE6/γTCP29)_140_) was prepared via introduction of hydrophobic γTCP groups onto the pendant VE groups by acetal linkage (Figure 1). The plane and subscript numbers express the content (mol%) and degree of polymerization (DP), respectively. In addition, aqueous solutions of PEG_54_-P(AA/VE6/γTCP29)_140_ were formulated to produce polymer micelles via hydrophobic interactions of pendant γTCP (Figure 2). Under acidic conditions, partial release of γTCP occurred due to the decomposition of the pendant acetal linkages. This was also attributed to the slight swelling of the polymer micelles since hydrophobicity of the micelles decreased with the release of hydrophobic γTCP. This suggests that the release of highly concentrated γTCP by the polymer micelles under acidic conditions may lead to cancer-cell apoptosis without the use of anticancer drugs.

## 2. Materials and Methods

### 2.1. Materials

Poly(ethylene glycol) methylether (4-cyano-4-pentanoate dodecyltrithiocarbonate) (PEG_54_, *M*_n_ = 2.40 × 10^3^ g/mol) and γ-tocopherol (γTCP, 96%) were purchased from Sigma Aldrich (St. Louis, MO, USA); 1-ethyl-3-(3-dimethylaminopropyl)carbodiimide hydrochloride (EDC, 98%) and *N*,*N*-dimethyl-4-aminopyridine (DMAP, 99%) were procured from Fujifilm Wako Pure Chemicals (Osaka, Japan). Ethylene glycol mono-vinyl ether (EGVE, 98%) and *p*-toluenesulfonic acid (*p*-TSA, 98%) were supplied by Tokyo Chemical Industry (Tokyo, Japan). All reagents above were used as received without further purification. We used 2,2′-azobisisobutyronitrile (AIBN, 98%) from Sigma Aldrich (St. Louis, MO, USA) after recrystallization using methanol. Acrylic acid (AA, 98%), 1,4-dioxane (99%), and *N*,*N*-dimethylformamide (DMF, 99%) from Fujifilm Wako Pure Chemicals (Osaka, Japan) were used after vacuum distillation. Water was purified with an ion-exchange column system. Other reagents were used as received.

### 2.2. PEG_54_-PAA_140_ Synthesis

PEG_54_ (2.00 g, 0.833 mmol), AA (9.00 g, 124 mmol), and AIBN (30.5 mg, 0.186 mmol) were dissolved in 1, 4-dioxane (50 mL). The solution was deoxygenated by purging argon gas for 30 min. Polymerization was carried out at 60 °C for 16 h. The conversion of AA estimated from ^1^H NMR was 98.2%. The polymerization mixture was dialyzed against pure water for 2 days. The polymer (PEG_54_-PAA_140_) was recovered by a freeze-drying method (8.70 g, 79.1%). DP for PAA block and number-average molecular weight (*M*_n_) for PEG_54_-PAA_140_ were estimated using ^1^H NMR as 140 and 1.26 × 10^4^ g/mol, respectively. While, *M*_n_ and molecular-weight distribution (*M*_w_/*M*_n_) were calculated as 1.25 × 10^4^ g/mol and 1.36, respectively, using gel-permeation chromatography (GPC).

### 2.3. Synthesis of PEG_54_-P(AA/VE35)_140_

We have used the conventional esterification method using EDC [26]. PEG_54_-PAA_140_ (134 mg, 1.50 mmol of COOH unit), DMAP (91.8 mg, 0.751 mmol), and EDC (860 mg, 4.49 mmol) were dissolved in DMF (20 mL). The solution was stirred at 30 °C for 10 h under an argon atmosphere, and then EGVE (0.667 mL, 7.55 mmol) was added to the solution. The solution was stirred at 30 °C for 24 h. Once the reaction was complete, the solution was dialyzed against pure water for three days. The prepared polymer (PEG_54_-P(AA/VE35)_140_) was recovered by a freeze-drying method (0.15 g, 56.6%). The *M*_n_ of PEG_54_-P(AA/VE35)_140_ estimated from ^1^H NMR was determined as 1.67 × 10^4^ g/mol. *M*_n_ and *M*_w_/*M*_n_ estimated using GPC were determined as 7.02 × 10^3^ g/mol and 1.38, respectively. The exchange ratio from the pendant carboxylic acid in the PAA block to the VE group was 35.0% estimated from ^1^H NMR.

### 2.4. PEG_54_-P(AA/VE6/γTCP29)_140_ Synthesis

Conjugation of γTCP onto PEG_54_-P(AA/VE35)_140_ via an acid-labile acetal bond was prepared according to a previously reported method [17]. PEG_54_-P(AA/VE35)_140_ (62.5 mg, 180 µmol of VE unit), γTCP (25.0 mg, 60.0 µmol), and *p*-TSA (0.56 mg, 2.94 µmol) were dissolved in DMF (10 mL). Molecular sieves 4A (1.00 g) was added to the solution. The reaction was carried out at 50 °C for 4 days under an argon atmosphere. Molecular sieves were removed by filtration, and the solution was dialyzed against pure water for 2 days. The polymer (PEG_54_-P(AA/VE6/γTCP29)_140_) was recovered by a freeze-drying method (80.9 mg, 92.5%). *M*_n_ for PEG_54_-P(AA/VE6/γTCP29)_140_ using ^1^H NMR was estimated as 1.96 × 10^4^ g/mol. *M*_n_ and *M*_w_/*M*_n_ estimated using GPC were determined as 1.02 × 10^4^ g/mol and 1.47, respectively. The exchange ratio from the VE to γTCP was calculated as 82.9% from ^1^H NMR.

### 2.5. Measurements

^1^H NMR spectra were measured with a Bruker DRX-500 (Billerica, AM, USA) at room temperature. The block copolymer sample solutions for the ^1^H NMR measurements were prepared in dimethylsulfoxide-*d*_6_ (DMSO-*d*_6_). GPC measurements were performed using Shodex (Tokyo, Japan) Asahipak GF-1G guard column and GF-7M HQ column at 40 °C with a refractive index detector and yielded a phosphate buffer solution (PBS) of pH 9 containing 10% *v*/*v* of acetonitrile at 0.6 mL/min in the developing solvent. Determination of *M*_n_ and *M*_w_/*M*_n_ according to GPC were calibrated using standard sodium poly(styrene sulfonate)s. Hydrodynamic radius (*R*_h_) and scattered light intensity (LSI) were estimated using a Malvern (Malvern, UK) Zetasizer Nano ZS-ZEN3600 equipped with a He–Ne laser source (4 mW at 632.8 nm) at 25 °C. The sample solutions for light-scattering measurements were filtered through a filter with pore-size diameter of 0.8 µm. Static light scattering (SLS) measurements were carried out with Otsuka Electronics (Osaka, Japan) DLS 7000 at 25 °C. The He–Ne laser (10 mW at 632.8 nm) was used as a light source. Weight average molecular weight (*M*_w_) and radius of gyration (*R*_g_) were calculated from a Debye plot at 1 polymer concentration. The Rayleigh ratio of toluene was used in instrument calibration. Refractive index increment against the polymer concentration (d*n*/d*C*_p_) at 633 nm was determined using Otsuka Electronics DRM-3000 differential refraction meter at 25 °C. TEM observations were performed with a JEOL (Tokyo, Japan) JEM-2100 at an accelerating voltage of 160 kV. Prior to TEM analysis, the sample was prepared by placing 1 drop of the aqueous solution on a copper grid coated with Formvar. Excess water was blotted using filter paper. Samples were stained by sodium phosphotungstate and dried under vacuum for a period of 1 day.

## 3. Results and Discussion

### 3.1. PEG_54_-P(AA/VE6/γTCP29)_140_ Preparation

**Figure 3:**
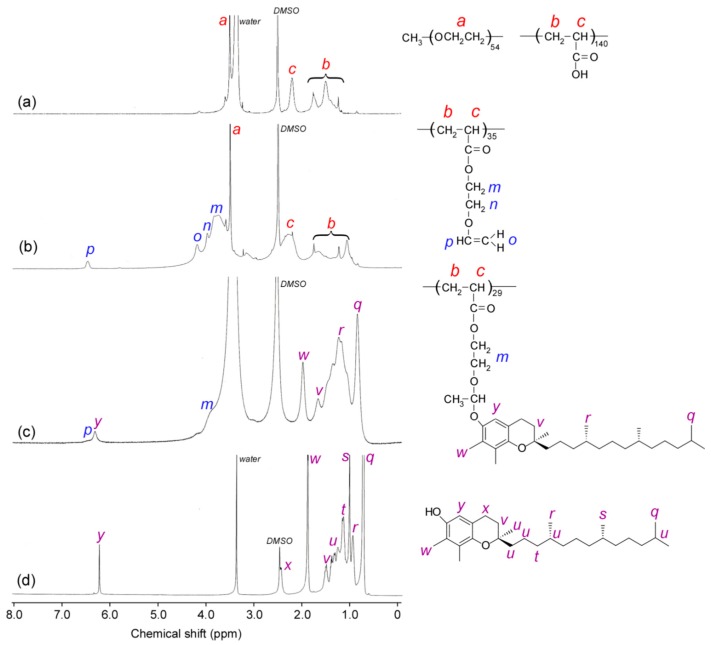
^1^H NMR spectra of (**a**) PEG_54_-PAA_140_, (**b**) PEG_54_-P(AA/VE35)_140_, (**c**) PEG_54_-P(AA/VE6/γTCP29)_140_, and (**d**) γTCP in DMSO-*d*_6_.

The conversion of AA by RAFT polymerization using PEG_54_ macro-CTA was determined as 98.2% estimated from the decrease of ^1^H NMR integral area intensity ratio of the vinyl group in AA after polymerization. The DP of the PAA block was calculated to be 140 from the area integral intensity ratio of peaks attributed to ethylene oxide protons in PEG_54_ at 3.7 ppm and the main chain protons in PAA at 2.3 ppm (Figure 3a). The introduction of the pendant vinyl groups in PEG_54_-P(AA/VE35)_140_ were confirmed by the observed ^1^H NMR signal at 6.5 ppm attributed to VE (Figure 3b). The reaction rate from the pendant carboxylic acid to the vinyl groups, calculated as 34.8%, was estimated from the integral intensity ratios of the attributed peaks to the main chain protons of PAA_140_ at 2.3 ppm and the vinyl protons in EGVE at 6.5 ppm. This result indicated that about 49 pendant vinyl groups were introduced into one polymer chain of PEG_54_-P(AA/VE35)_140_. The protons attributed to the terminal methyl at 0.8 ppm, benzyl at 2.0 ppm, and phenyl protons at 6.3 ppm in the pendant γTCP could be observed from a PEG_54_-P(AA/VE6/γTCP29)_140_^1^H NMR spectrum (Figure 3c). The introduction rate of γTCP in PEG_54_-P(AA/VE6/γTCP29)_140_ was calculated as 83.3% estimated from the integral intensity ratio of the unreacted pendant vinyl protons in PEG_54_-P(AA/VE35)_140_ at 6.5 ppm and phenyl protons in γTCP at 6.3 ppm [27]. This result indicated that about 41 pendant γTCPs were introduced into a single polymer chain of PEG_54_-P(AA/VE6/γTCP29)_140_. The theoretical *M*_n_ of each polymer was calculated using the following equation:

(1) $$M_{n}\left(\mathrm{theo}\right)=\frac{{\left[M\right]}_{0}}{{\left[\mathrm{CTA}\right]}_{0}}\times \frac{p}{100}\times M_{m}+M_{\mathrm{CTA}},$$

where [M]_0_ and [CTA]_0_ are the initial molar concentrations of monomer and CTA, respectively, *p* is the conversion of the monomer, and *M*_m_ and *M*_CTA_ are the molecular weights of the monomer and CTA, respectively.

**Figure 4:**
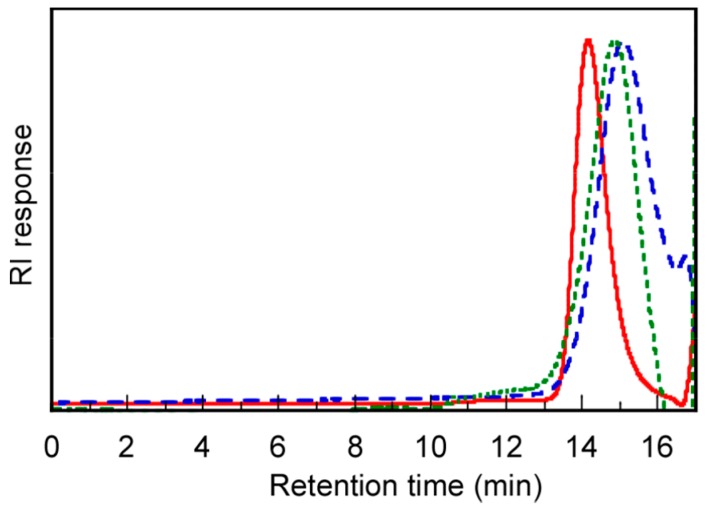
Gel-permeation chromatography (GPC) elution curves of PEG_54_-PAA_140_ (―), PEG_54_-P(AA/VE35)_140_ (– –), and PEG_54_-P(AA/VE6/γTCP29)_140_ (----) at 40 °C using a phosphate buffer solution (PBS) at pH 9 containing 10% *v*/*v* acetonitrile as the eluent.

The GPC-determined *M*_n_ values of PEG_54_-PAA_140_, PEG_54_-P(AA/VE35)_140_, and PEG_54_-P(AA/VE6/γTCP29)_140_ were 1.25 × 10^4^, 7.02 × 10^3^, and 1.02 × 10^4^ g/mol, respectively (Figure 4). Due to the unexpected interactions between polymers within the GPC column, and the difference between structures of the measured polymer and the standard polymer, accurate molecular weight could not be estimated [28,29]. The *M*_w_/*M*_n_ values of PEG_54_-PAA_140_, PEG_54_-P(AA/VE35)_140_, and PEG_54_-P(AA/VE6/γTCP29)_140_ were relatively narrow, obtained as 1.36, 1.38, and 1.47, respectively. Table 1 summarizes the molecular weight and *M*_w_/*M*_n_ of each polymer.

### 3.2. PEG_54_-P(AA/VE6/γTCP29)_140_ Micelle Formation

**Figure 5:**
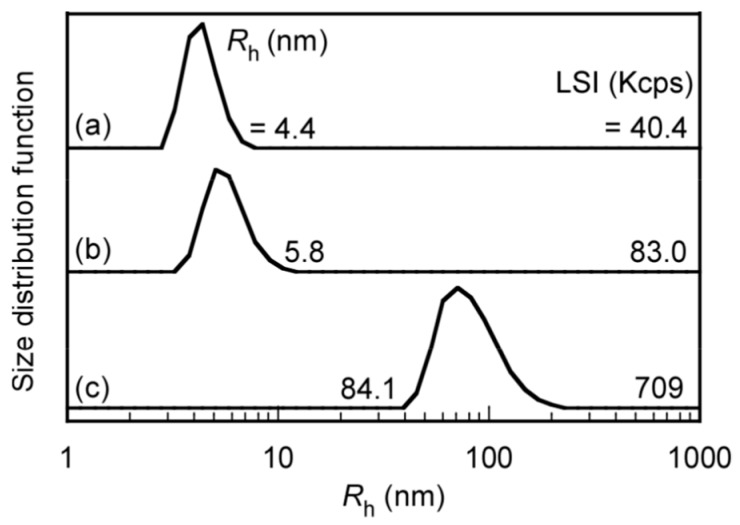
Hydrodynamic-radius (*R*_h_) distributions for (a) PEG_54_-PAA_140_, (b) PEG_54_-P(AA/VE35)_140_, and (c) PEG_54_-P(AA/VE6/γTCP29)_140_ in PBS at *C*_p_ = 0.10 g/L.

The *R*_h_ and LSI of the polymers were measured at *C*_p_ = 0.10 g/L in a PBS buffer of pH 7.4 at 25 °C to confirm aggregation-state changes for PEG_54_-PAA_140_, PEG_54_-P(AA/VE35)_140_, and PEG_54_-P(AA/VE6/γTCP29)_140_ (Figure 5). The *R*_h_ values of PEG_54_-PAA_140_ and PEG_54_-P(AA/VE35)_140_ were 4.4 and 5.8 nm, respectively. The LSI values of PEG_54_-PAA_140_ and PEG_54_-P(AA/VE35)_140_ were estimated as 40.4 and 83.0 Kcps, respectively. These polymers may dissolve in PBS to form unimers due to their small *R*_h_ and LSI values. On the other hand, the *R*_h_ and LSI of PEG_54_-P(AA/VE6/γTCP29)_140_ increased to 84.1 nm and 709 Kcps, suggesting the formation of polymer micelles. These polymer micelles were formed due to the hydrophobic interaction between pendant γTCP groups, which were composed of a hydrophobic P(AA/VE6/γTCP29)_140_ core and hydrophilic PEG_54_ shells. The end-to-end distance of the PEG_54_-P(AA/VE6/γTCP29)_140_ polymer chain was estimated as 53.4 nm, assuming that the lengths of the vinyl monomer unit and the PEG chain were 0.25 and 18.4 nm, respectively [30,31,32]. The *R*_h_ value of the polymer micelle was calculated as 84.1 nm, which was larger than the end-to-end distance of PEG_54_-P(AA/VE6/γTCP29)_140_ (=53.4 nm). Therefore, polymers could not form simple spherical core–shell structures. The polymers may form intermicellar aggregates or large compound micelles.

**Figure 6:**
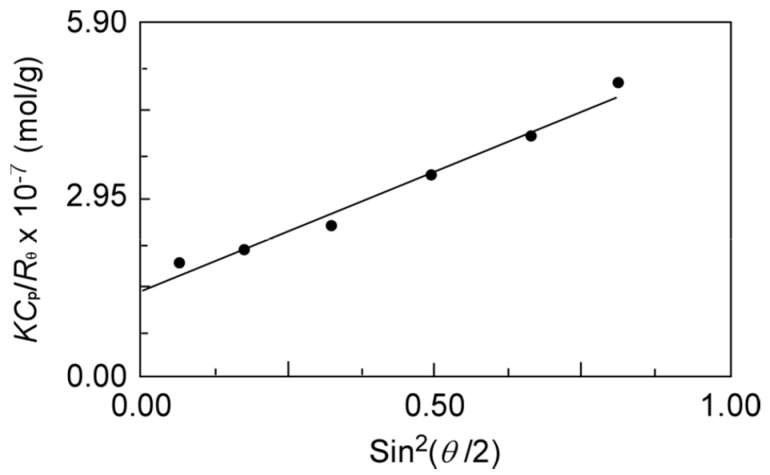
Debye plot for PEG_54_-P(AA/VE6/γTCP29)_140_ in PBS at fixed *C*_p_ (=0.10 g/L). *K*, optical constant; *C*_p_, polymer concentration; *R*_θ_, difference between Rayleigh ratio of solution and solvent; and *θ*, scattering angle.

To characterize the polymer micelles formed from PEG_54_-P(AA/VE6/γTCP29)_140_ in the PBS buffer, SLS measurements were performed (Figure 6). Using SLS, the apparent *M*_w_ and *R*_g_ were estimated from the Debye plot (Table 2). The measured d*n*/d*C*_p_ (=1.70 mL/g) of the polymer micelles were then used to estimate the apparent *M*_w_. The aggregation number (*N*_agg_), which is the number of polymer chains to form one micelle, was estimated using the following equation:

(2) $$N_{\mathrm{agg}}=\frac{M_{w}(\mathrm{SLS})}{M_{w}/M_{n}\times M_{n}(\mathrm{NMR})}$$

*N*_agg_ was estimated as 248 using *M*_w_ (=7.15 × 10^6^ g/mol), *M*_w_/*M*_n_ (=1.47), and *M*_n_(NMR) (=1.96 × 10^6^ g/mol) determined by SLS. Theoretically, *R*_g_/*R*_h_ depends on the shape and polydispersity of the micelles, for instance, hard sphere as 0.775, monodisperse sphere as 1.0, and rodlike shape as more than 2.0 [33,34,35]. The *R*_g_/*R*_h_ of the polymer micelles formed from PEG_54_-P(AA/VE6/γTCP29)_140_ was calculated as 1.32, which is close to 1.0. Therefore, the polymers formed spherical micelles.

### 3.3. Micelle Decomposition in Acidic Condition

**Figure 7:**
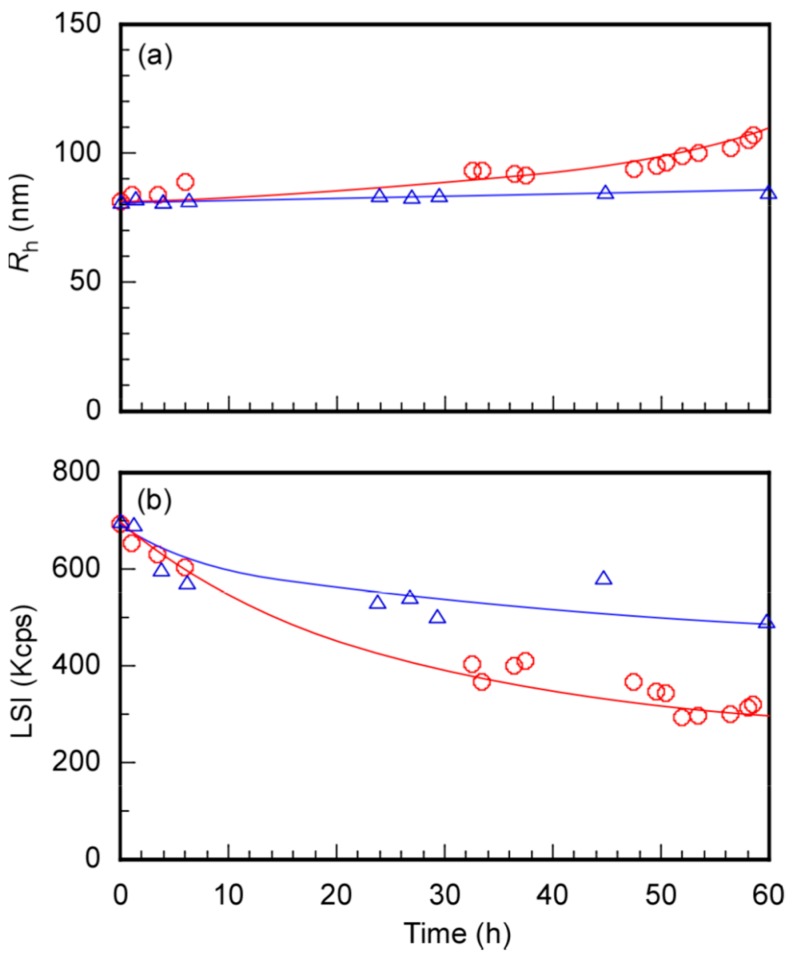
Time dependence on (**a**) hydrodynamic radius (*R*_h_) and (**b**) light-scattering intensity (LSI) for PEG_54_-P(AA/VE6/γTCP29)_140_ at *C*_p_ = 0.10 g/L at 25 °C in acetate buffer of pH 5.2 (○) and in PBS buffer of pH 7.4 (△).

To evaluate the time dependence on micelle size and density formed from PEG_54_-P(AA/VE6/γTCP29)_140_ in acetate buffer of pH 5.2 (0.01 M), *R*_h_ and LSI changes were monitored at 25 °C (Figure 7). At constant particle size, density depended on the LSI. As a reference experiment, the time dependence on *R*_h_ and LSI of the polymer micelles in a PBS buffer of pH 7.4 (0.01 M) were also monitored. *R*_h_ increased with increasing time at pH 5.2. *R*_h_ values just after preparation and after 60 h at pH 5.2 were determined as 81.0 and 107.0 nm, respectively. LSI values of just after preparation and after 60 h at pH 5.2 were estimated as 693 and 321 Kcps, respectively. The LSI were observed to decrease with increasing time. At pH 5.2, the pendant acetal bonds decomposed to release the hydrophobic γTCP from the polymer micelles. As a result, hydroxyl groups were generated at the pendant groups. However, not all pendant acetal bonds were decomposed at pH 5.2. However, an increase in *R*_h_ and decrease of the LSI was due to decreasing densities observed at pH 5.2, attributed to the hydration and then swelling of the core of the polymer micelles. Oho et al. [36] reported that γTCP is relatively stable against acidic conditions. The released γTCP from the polymer in a buffer at pH = 5.2 may not be decomposed.

At pH 7.4, the *R*_h_ values of the micelles just after preparation and after 60 h were calculated as 81.5 and 83.8 nm, respectively. The LSI of the micelles slightly decreased with increasing time. The values of LSI just after preparation and after 60 h were obtained as 695 and 492 Kcps. These observations suggest that the pendant acetal groups slightly decomposed at pH 7.4. Compared to the time dependence of *R*_h_ and LSI at pH 5.2 and 7.4, γTCP was effectively released due to the decomposition of the pendant acetal bonds.

**Figure 8:**
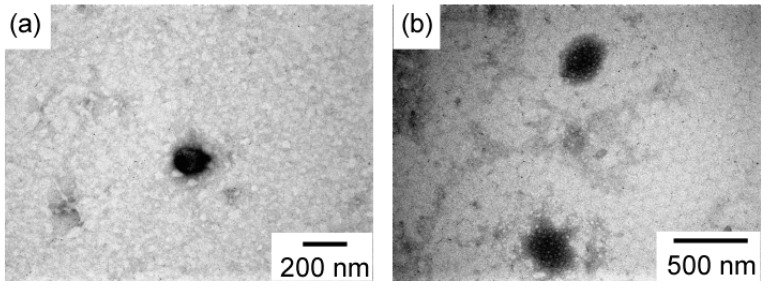
Transmission electron microscopy (TEM) images of PEG_54_-P(AA/VE6/γTCP29)_140_ at *C*_p_ = 0.10 g/L in acetate buffer of pH 5.2 (**a**) just after preparation and after (**b**) 60 h.

The micelles of PEG_54_-P(AA/VE6/γTCP29)_140_ in PBS of pH 7.4 were observed with Transmission electron microscopy (TEM, Figure S1). The radius (*R*_TEM_) estimated from the TEM image was 72.1 ± 12 nm. *R*_TEM_ was calculated from a randomly selected 50 particles (*N* = 50). *R*_TEM_ was smaller than the *R*_h_ values determined by light-scattering measurements because the aggregates shrank during the drying process in preparation for TEM measurements. The micelles of PEG54-P(AA/VE6/γTCP29)140 in the acetate buffer of pH 5.2 just after preparation and after 60 h were observed using TEM to confirm the shape changes of the micelles (Figure 8 and Figure S1). Both samples were close to spherical shapes. The *R*_TEM_ values for just after preparation and after 60 h were estimated as 77.9 ± 18 and 113 ± 32 nm, respectively. This result suggests that the swollen micelles were due to increasing core hydrophilicity at pH 5.2 after 60 h. The particle-size range of the polymer aggregates after 60 h was larger than that just after preparation. This observation suggests that decomposition of the pendant acetal linkage was not uniform.

## 4. Conclusions

The amphiphilic block copolymers of PEG_54_-P(AA/VE6/γTCP29)_140_ bearing hydrophobic γTCP via acetal bond linkage were prepared using RAFT radical polymerization. PEG_54_-P(AA/VE6/γTCP29)_140_ were demonstrated to dissolve in water, forming polymer micelles composed of hydrophobic P(AA/VE6/γTCP29)_140_ block cores and hydrophilic PEG_45_ shells. At pH 5.2, a gradual increase in micelle size was observed with the gradual increase in time, attributed to the hydration and swelling of the core, which leads to the breakage of pendant acetal bonds. The breaking of acetal bonds generated γTCP and pendant hydrophilic hydroxyl groups, which were hydrated and swollen to increase micelle size. At pH 7.4, the LSI of the polymer aqueous solutions decreased slightly with increasing time. This observation suggested that acetal bond was slightly degraded, even at pH 7.4. By applying PEG_54_-P(AA/VE6/γTCP29)_140_ as a suitable polymer for cancer treatment, a slow release of γTCP could be achieved. However, low concentrations of γTCP show nontoxicity, as well as no side effects for healthy tissue; when γTCP is released in large amounts around cancer tissue, pendant acetal linkages are effectively broken due to low pH values. Moreover, the high released concentrations of γTCP from the polymer micelle present an alternative route in the treatment of cancer tissue in the absence of anticancer-drug usage, especially prostate cancer, with reduced side effects.

## Figures and Tables

**Table 1:** Number-average molecular weight (*M*_n_) and *M*_n_ distribution (*M*_w_/*M*_n_).

Sample	Theoretical *M*_n_ × 10^4^ (g/mol)	^1^H NMR *M*_n_ × 10^4^ (g/mol)	GPC *M*_n_ × 10^4^ (g/mol)	*M*_w_/*M*_n_
PEG_54_-PAA_140_	1.30	1.26	1.25	1.36
PEG_54_-P(AA/VE35)_140_	1.54	1.67	0.702	1.38
PEG_54_-P(AA/VE6/γTCP29)_140_	1.83	1.96	1.02	1.47

**Table 2:** Dynamic and static light-scattering data of PEG_54_-P(AA/VE6/γTCP29)_140_ in PBS.

Sample	SLS *M*_w_ × 10^6^ (g/mol)	*N*_agg_	*R*_g_ (nm)	*R*_h_ (nm)	*R*_g_/*R*_h_	d*n*/d*C*_p_ (mL/g)
PEG_54_-P(AA/VE6/γTCP29)_140_	7.15	248	111	84.1	1.32	1.70

## Supplementary Materials

The following are available online at https://www.mdpi.com/2073-4360/12/1/36/s1: Figure S1. (a) TEM images of PEG_54_-P(AA/VE6/γTCP29)_140_ at *C*_p_ = 0.10 g/L in PBS buffer of pH 7.4, in acetate buffer of pH 5.2 (b) just after preparation and (c) after 60 h.

## References

[1] Lefèvre N., Fustin C.A., Gohy J.F. (2009). Polymeric micelles induced by interpolymer complexation. Macromol. Rapid Commun..

[2] Zhang L., Eisenberg A. (1996). Ion-induced morphological changes in “crew-cut” aggregates of amphiphilic block copolymers. Science.

[3] Nakashima K., Bahadur P. (2006). Aggregation of water-soluble block copolymers in aqueous solutions: Recent trends. Adv. Colloid Interface Sci..

[4] Yokoyama M., Okano T., Sakurai Y., Kataoka K. (1994). Improved synthesis of adriamycin-conjugated poly(ethylene oxide)-poly(aspartic acid) block copolymer and formation of unimodal micellar structure with controlled amount of physically entrapped Adriamycin. J. Control. Release.

[5] Cabral H., Matsumoto Y., Mizuno K., Chen Q., Murakami M., Kimura M., Terada Y., Kano M.R., Miyazono K., Uesaka M. (2011). Accumulation of sub-100 nm polymeric in poorly permeable tumors depends on size. Nat. Nanotechnol..

[6] Lammers T., Subr V., Ulbrich K., Peschke P., Huber P.E., Hennink W.E., Storm G. (2009). Simulataneous delivery of doxorubicin and gemcitabine to tumors in vivo using prototypic polymeric drug carriers. Biomaterials.

[7] Matsumura Y., Maeda H. (1986). A new concept for macromolecular therapeutics in cancer chemotherapy: Mechanism of tumoritropic accumulation of proteins and the antitumor agent smancs. Cancer Res..

[8] Deng C., Jiang Y.J., Cheng R., Meng F.H., Zhong Z.Y. (2012). Biodegradable polymeric micelles for targeted and controlled anticancer drug delivery: Promiss, progress and prospects. Nano Today.

[9] Hatakeyama H., Akita H., Harashima H. (2011). A multifunctional envelope type nano device (MEND) for gene delivery to tumours based on the EPR effect: A strategy for overcoming the PEG dilemma. Adv. Drug Deliv. Rev..

[10] Hsiao C., Hsiao G., Chen W., Wang S., Chiang C., Liu L., Guh J., Lee T., Chung C. (2014). Cephalochromin induces G0/G1 cell cycle arrest and apoptosis in A549 human non-small-cell lung cancer cells by inflicting mitochondrial disruption. J. Nat. Prod..

[11] Kumazoe M., Sugihara K., Tsukamoto S., Huang Y., Tsurudome Y., Suzuki T., Suemasu Y., Ueda N., Yamashita S., Kim Y. (2013). 67-kDa laminin receptor increases cGMP to induce cancer-selective apoptosis. J. Clin. Investig..

[12] Jiang Q., Wong J., Fyrst H., Saba J.D., Ames B.N. (2004). γ-Tocopherol or combinations of vitamin E forms induce cell death in human prostate cancer cells by interrupting sphingolipid synthesis. Proc. Natl. Acad. Sci. USA.

[13] Heinonen O., Albanes D., Vittamo J., Taylor P., Huttunen J., Hartman A., Haapa-koski J., Malila N., Rautalahti M., Ripatti S. (1998). Prostate cancer and supplementation with *α*-tocopherol and *β*-tocopherol. J. Natl. Cancer Inst..

[14] Noomura A., Stemmermann G., Lee J. (1997). Serum micronutrients and prostate cancer in Japanese Americans in Hawaii. Cancer Epidemiol. Prev. Biomark..

[15] Blomstrand R., Forsgren L. (1968). Labelled tocopherols in man. Int. Z. Vitaminforschung.

[16] Coleman C.N., Mitchell J.B., Camphausen K. (2002). Tumor hypoxia: Chicken, egg, or a piece of the farm?. J. Clin. Oncol..

[17] Gu Y., Zhong Y., Meng F., Cheng R., Deng C., Zhong Z. (2013). Acetal-linked paclitaxel prodrug micellar nanoparticles as a versatile and potent platform for cancer therapy. Biomacromolecules.

[18] Wei H., Zhuo R.X., Zhang X.Z. (2012). Design and development of polymeric micelles with cleavable links for intracellular drug delivery. Prog. Polym. Sci..

[19] Gillies E.R., Jonsson T.B., Frechet J.M.J. (2004). Stimuli-responsive supramolecular assemblies of linear-dendritic copolymers. J. Am. Chem. Soc..

[20] Gillies E.R., Frechet J.M.J. (2005). PH-responsive copolymer assemblies for controlled release of doxorubicin. Bioconjug. Chem..

[21] Chen W., Meng F.H., Cheng R., Zhong Z.Y. (2010). PH-sensitive degradable polymersomes for triggered release of anticancer drug: A comparative study with micelles. J. Control. Release.

[22] Simon H., Lien H., Benoit L., Lien L., Ruben C., Sabah K., Aaron E., Sunil D., Lutz N., Bert S. (2018). Transiently thermoresponsive acetal polymers for safe and effective administration of anphotericin B as a vaccine adjuvant. Bioconjug. Chem..

[23] Lampen J. (1969). Amphotericin B and other polyenic antifungal antibiotics. Am. J. Clin. Pathol..

[24] Gold W., Stout H.A., Pagano J.F., Donovick R. (1955). Amphotericins A & B, antifungal produced by a streptomycete. I. In vitro studies. Antibitics Ann..

[25] Feng L., Chao C., Xixi Y., Xinyu H., Zhangyan Z., Jie L., Yue Y., Xianzhu Y., Jun W. (2018). Acetal-linked hyperbranched polyphosphoester nanocarriers loaded with chlorin e6 for pH-activatable photodynamic therapy. ACS Appl. Mater. Interfaces.

[26] Kang J., Zhang X.Y., Sun L.D., Zhang X.X. (2007). Bioconjugation of functionalized fluorescent YVO_4_: Eu nanocrystals with BSA for immunoassay. Talanta.

[27] Ng M.H., Choo Y.M., Ma A.N., Chuah C.H., Hashim M.A. (2004). Separation of vitamin E (tocopherol, tocotrienol, and tocomonoenol) in palm oil. Lipids.

[28] Hadjichristidis N., Fetters L.J. (1982). Effect of molecular weight and chain branching on the refractive index increment of polystyrene and polyisoprene solutions. J. Polym. Sci. Polym. Phys. Ed..

[29] Binboga N., Kisakurek D., Baysal B.M. (1985). Effect of molecular weight on the refractive index increments of polystyrene, poly(ethylene glycol), poly(propylene glycol), and poly(dichlorophenylene oxide) in solution. J. Polym. Sci. Polym. Phys. Ed..

[30] Yamamoto Y., Nagasaki Y., Kato Y., Sugiyama Y., Kataoka K. (2001). Long-circulating poly(ethylene glycol)–poly(D,L-lactide) block copolymer micelles with modulated surface charge. J. Control. Release.

[31] Tanford C., Nozaki Y., Rohde M.F. (1977). Size and shape of globular micelles formed in aqueous solution by *n*-alkyl polyoxyethylene ethers. J. Phys. Chem..

[32] Yusa S., Yokoyama Y., Morishima Y. (2009). Synthesis of oppositely charged block copolymers of poly(ethylene glycol) via reversible addition-fragmentation chain transfer radical polymerization and characterization of their polyion complex micelles in water. Macromolecules.

[33] Burchard W. (1983). Static and dynamic light scattering from branched polymers and biopolymers. Adv. Polym. Sci..

[34] Konishi T., Yoshizaki T., Yamakawa H. (1991). On the “universal constants” *ρ* and *ϕ* of flexible polymers. Macromolecules.

[35] Akcasu Z.A., Han C.C. (1979). Molecular weight and temperature dependence of polymer dimensions in solution. Macromolecules.

[36] Oho H., Yu T., Xu L.X. (1995). Effects of several tea components on acid resistance of human tooth enamel. J. Dent..

