# Supplementary material for: Amphiphilic Block Copolymers Bearing Hydrophobic γ-Tocopherol Groups with Labile Acetal Bond
Source: Polymers (Basel). 2019 Dec 25;12(1):36. doi: 10.3390/polym12010036 (PMC7023524; doi:10.3390/polym12010036)
Supplement: Supplementary file 1 [file polymers-12-00036-s001.pdf]

Supplementary Materials

Amphiphilic Block Copolymer Bearing Hydrophobic γ-Tocopherol Groups with Labile Acetal Bond

Shotaro Yukioka [1], Takuya Kitadume [2], Suchismita Chatterjee [2], Gan Ning [2], Tooru Ooya [2] and Shin-ichi Yusaa [1,*]

[1]Graduate School of Engineering, University of Hyogo, 2167 Shosha, Himeji, Hyogo 671-2280, Japan

[2]Graduate School of Engineering, Kobe University, 1-1 Rokkoudai, Nada, Kobe, Hyogo 657-8501, Japan

*Correspondence: yusa@eng.u-hyogo.ac.jp

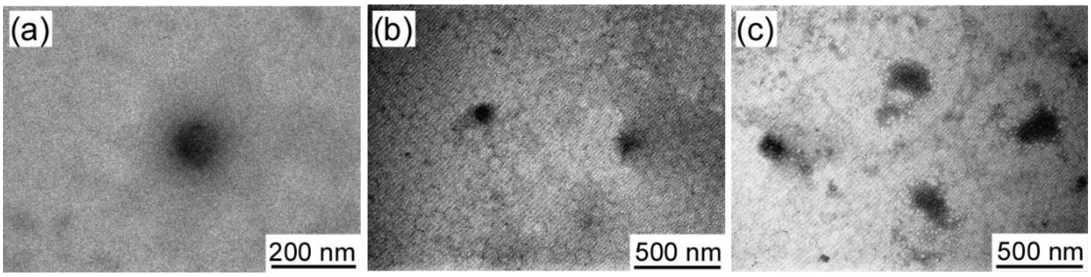

Figure S1. TEM images of PEG$_{54}$-P(AA/VE6/γTCP29)$_{140}$ at C_P = 0.10 g/L in PBS buffer of pH 7.4 (a), in acetate buffer of pH 5.2 just after preparation (b) and after 60 h (c).
